# Multicellular senescence programs in the aged heart

**DOI:** 10.1016/j.jmccpl.2026.100852

**Published:** 2026-05-16

**Authors:** Laurent Bultot, Natsuko Tsurudome, Emi Kumazaki, Ippei Shimizu

**Affiliations:** a Department of Cardiovascular Aging, National Cerebral and Cardiovascular Center Research Institute, Osaka, 564-8565, Japan; b Department of Cardiovascular Medicine, National Cerebral and Cardiovascular Center, Osaka, 564-8565, Japan

**Keywords:** Cellular senescence, Heart, SASP, Senolysis

## Abstract

Cardiac aging reflects a convergence of intrinsic molecular damage and maladaptive stress responses that progressively erode myocardial resilience. Accumulating genomic instability, telomere dysfunction, chromatin remodeling, and metabolic dysregulation activate innate immune signaling and cellular senescence across cardiomyocytes, endothelial cells, fibroblasts, and immune cells. At the tissue level, these processes manifest as microvascular rarefaction, fibrosis, hypertrophy, neurovascular uncoupling, and impaired adaptive capacity, creating a substrate that overlaps extensively with cardiomyopathy, heart failure, and atrial fibrillation (AF).

Importantly, senescence in the heart is not monolithic. Emerging multi-omics and spatial analyses reveal context-dependent senescence programs, including transient, injury-associated states that support angiogenesis and repair, alongside chronic senescent phenotypes that propagate inflammation and remodeling through the senescence-associated secretory phenotype (SASP). These observations indicate that senescence is not uniformly deleterious but rather comprises functionally heterogeneous responses within the aging myocardium. Non-cell autonomous interactions-spanning cardiomyocyte–fibroblast crosstalk, immune niche signaling, endothelial cell-neuronal axis, and systemic organ-to-heart communication-further amplify or constrain these trajectories. Advances in biomarker discovery, imaging, and circulating epigenetic signatures now enable biological aging of the heart to be quantified beyond chronological aging, although many circulating biomarkers are not cardiac-specific and may also reflect systemic inflammation, fibrosis, frailty, or generalized biological aging. In parallel, preclinical studies demonstrate that senolytic, senomorphic, metabolic, and nutrient-sensing–targeted interventions can partially restore cardiac homeostasis. Together, these insights suggest that cellular senescence may represent a key mechanism and a potentially targetable process in cardiac aging, with important implications for the prevention and treatment of age-related cardiovascular disease.

## Graphical abstract

**Figure:**
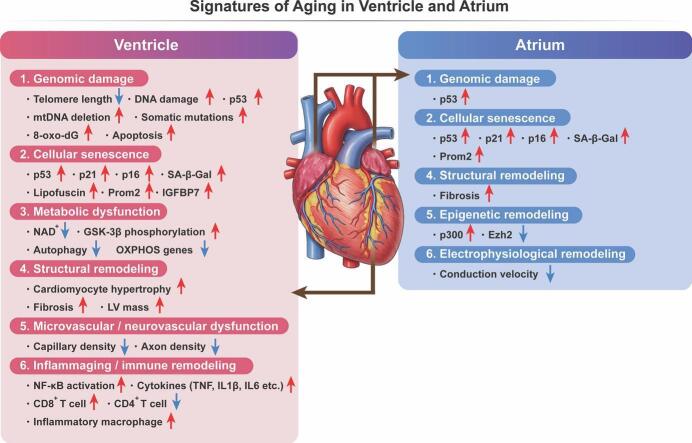

## 1 Introduction

Cardiac aging shares core molecular features with human cardiomyopathies, underscoring its direct relevance to clinical disease. Telomere shortening, deoxyribonucleic acid (DNA) damage, accumulation of senescent cells, canonical/non-canonical SASP factors, reactive oxygen species (ROS), and chronic sterile inflammation progressively accumulate in the aged heart. Importantly, similar signatures are observed in human and experimental cardiomyopathies. While both hypertrophic cardiomyopathy (HCM) and ischemic cardiomyopathy (ICM) are associated with shorter telomere length in cardiomyocytes [1], studies in dilated cardiomyopathy (DCM) have reported more variable findings regarding telomere dynamics [1], [2]. Notably, HCM is further characterized by elevated levels of DNA damage in cardiomyocytes [1]. These observations further suggest that aging-associated mechanisms may be differentially engaged across cardiomyopathy subtypes, highlighting the need for disease-contextualized interpretation. In pressure-overload models, p53 accumulation is associated with capillary rarefaction and cardiac dysfunction [3], [4], [5], consistent with aging-associated microvascular loss. Senescence-associated secretory phenotype (SASP)-related mediators are also detectable in failing hearts, with insulin-like growth factor-binding protein 7 (IGFBP7) elevated in cardiac tissue and in the circulation in heart failure, and its inhibition attenuates pressure overload-induced dysfunction [6], while broader panels of circulating SASP-linked proteins are being proposed as candidates for heart failure biomarkers in human studies [7]. Taken together, these observations suggest that dissecting senescence and aging mechanisms is relevant to understanding and potentially informing strategies to modulate the biology that underpins age-related cardiac diseases.

Given these shared aging-associated programs, it is becoming increasingly evident that the ventricle and atrium exhibit both common and chamber-specific senescence phenotypes. In this review, we discuss age-related mechanisms in the ventricle and atrium, highlighting their relative importance and the differences between cardiac chambers. In the following sections, we first characterize aging-related remodeling in the ventricle, followed by a focused discussion of the distinct biological features that define atrial aging.

## 2 Characterization of the aged ventricle

Several biological programs that define cardiac aging overlap with those activated during pathological remodeling. Cumulative molecular injury accumulates in the aged myocardium. This includes chromatin disorganization, DNA damage responses, mitochondrial dysfunction, chronic inflammation, metabolic dysregulation, suppression of autophagy and proteostasis, and senescence across cardiomyocyte and non-myocyte compartments [8], [9], [10], [11], [12], [13], [14], [15], [16], [17]. These contribute to progressive functional decline and reduced stress resilience [8], [9], [10], [11], [12], [13], [14], [15], [16], [17], as demonstrated in human and experimental studies, with these findings summarized in several reviews [18], [19], [20]. Tissue-level hallmarks consistently include capillary rarefaction, diminished nerve density, interstitial fibrosis, cardiomyocyte hypertrophy, increased apoptosis, and reduced proliferative/renewal capacity, collectively associated with reduced myocardial reserve and increased susceptibility to hemodynamic and metabolic stress [21], [22], [23], [24], as summarized in prior reviews [19], [25].

Several pieces of evidence have shown that metabolic stress is a key component of the aging process in the heart. Indeed, autophagy declines in the aged heart, partly mediated by reduced Atg9b-mediated autophagosome formation [12]. In addition to this, glycogen synthase kinase-3 beta (GSK-3β) enhances autophagy [26], while its function is suppressed in the phosphorylated form (pGSK-3β). The aged heart exhibits elevated levels of pGSK-3β [14], and constitutively active GSK-3β enhances autophagy in the aged heart, ameliorates systolic cardiac dysfunction, and improves survival in preclinical models [14]. Age-associated systemic metabolic alterations also influence cardiomyocyte biology, with aging associated with increased circulating phenylalanine levels, cyclin-dependent kinase inhibitor 1A (p21) expression, and epigenetic remodeling in cardiomyocytes [27], [28]. In parallel, nicotinamide adenine dinucleotide (NAD^+^), a key regulator of mitochondrial homeostasis and genomic stability, declines with aging both systemically and within the heart, and reduced NAD^+^ availability is linked to telomere instability and impaired DNA repair [29], [30], [31], [32]. In the aging heart, molecular imaging of cardiac sympathetic neuronal function demonstrates age-associated functional remodeling in vivo [33], [34], thus extending cardiac phenotyping beyond conventional structural assessments. In a longitudinal positron emission tomography (PET) study of aged rats, cardiac 11C-hydroxyephedrine uptake, an index of norepinephrine transporter function, declines with aging [34]. 123I-metaiodobenzylguanidine (MIBG) imaging demonstrates that inferior wall MIBG uptake diminishes with age in individuals without cardiac disease [33], indicating an age-related loss of cardiac sympathetic innervation [33], [34]. Recent studies demonstrate that aging reshapes the cardiac microenvironment in a region-specific manner, particularly within perivascular niches, with the primary hotspot characterized by an enrichment of senescent cells, activated fibroblasts, and inflammatory Lyve1^−^ macrophages interacting via the C3–C3ar1 axis [35]. Importantly, senescence in the heart does not present as a uniform pathology. Recent multi-omics analyses in adult mouse hearts have identified a transient, injury-associated senescence program regulated by Early growth response 1 (Egr1), which is linked to angiogenesis, cardiomyocyte proliferation, and extracellular matrix remodeling during cardiac repair. These findings suggest that senescence may exert context-dependent roles during cardiac injury and repair, indicating that senescence is not uniformly deleterious and highlighting the functional heterogeneity of senescent responses within the myocardium [36].

Building on these observations, the following sections examine how aging and cellular senescence are manifested across the major cellular constituents of the ventricle, including cardiomyocytes, endothelial cells, fibroblasts, and immune cells.

## 3 Cellular senescence in ventricular cardiomyocytes

### 3.1 Initiation of cardiomyocyte senescence: DNA damage, stress signaling, and transcriptional reprogramming

Increasing evidence indicates that cardiomyocyte senescence results from convergent cellular stresses that begin during postnatal maturation and progressively become intensified with aging, as demonstrated in human and experimental studies. Sarcomere assembly and increased oxidative metabolism are associated with increased ROS generation, which contributes to DNA damage and activation of p53, driving cell-cycle arrest and polyploidization in human cardiomyocytes [37]. In parallel, postnatal telomere shortening and dysfunction promote p21-dependent cell-cycle exit and binucleation, sharply restricting regenerative capacity after birth, although this remains reversible through p21 suppression [38]. These early barriers are further reinforced by p53-murine double minute 2 homolog (MDM2)-dependent microRNA (miRNA) networks that stabilize the postmitotic state, while its disruption allows cardiomyocyte cell-cycle re-entry but results in dilated cardiomyopathy, underscoring its role in cardiac homeostasis [39].

With aging, cardiomyocytes progressively accumulate persistent genomic injury, detected as telomere-associated DNA damage foci (TAF) [40]. Notably, cardiomyocytes isolated from aged myocardia exhibit elevated p21 protein expression, increased SA-β-Gal activity, and upregulation of *Cdkn1a* and *Cdkn2a* transcripts [40]. In addition, these aged cardiomyocytes acquire a non-canonical senescence-associated secretory phenotype, characterized by increased production of factors, e.g., growth differentiation factor 15 (GDF15), transforming growth factor-β2 (TGF-β2), and endothelin-3 (EDN3), thereby influencing the myocardial microenvironment [40]. Transcriptional reprogramming is also shown to further consolidate senescence, with p53-induced prominin 2 (Prom2) linking hypertrophic remodeling to the senescent phenotype [41].

Cell-intrinsic stress pathways further amplify and stabilize cardiomyocyte senescence; while loss of proprotein convertase subtilisin/kexin type 6 (PCSK6) has been shown to predispose cardiomyocytes toward a DNA damage-inducible transcript 3 (DDIT3)-associated endoplasmic reticulum (ER) stress response, accompanied by increased oxidative stress and p16/p21 activation, whereas PCSK6 restoration has been demonstrated to attenuate these changes [42]. An aged heart exhibits a diminished inhibition of NF-κB alpha (IκBα), as well as an increase in TNFα and nuclear factor kappa-light-chain-enhancer of activated B cells (NF-κB) p65 (RelA) [43]; it is also associated with elevated levels of calcium/calmodulin-dependent protein kinase II (CaMKII) [44]. Persistent activation of CaMKII promotes NF-κB-dependent SASP production and senescence, whereas its inhibition suppresses these pro-aging responses [44]. Notably, NF-κB signaling is shown to be context-dependent in cardiomyocytes, with sustained nuclear activity accelerating senescence, and its complete cardiomyocyte-specific suppression disrupts homeostasis and worsens remodeling in preclinical models [45]. In addition to these stress-responsive pathways, epigenetic and RNA-based regulators further modulate cardiomyocyte senescence susceptibility through chromatin remodeling and post-transcriptional control, including myelodysplasia/myeloid leukemia factor 1 (MLF1)-E1A binding protein p300 (EP300)-dependent epigenetic regulation, and microRNA-mediated stress responses [46], [47]. Together, these findings indicate that cardiomyocyte senescence is a progressive phenomenon governed by a multi-layered regulatory network integrating stress signaling, transcriptional control, and epigenetic remodeling. Elucidating these mechanisms will be essential for identifying modulators of senescence initiation and for targeting the molecular basis of cardiac aging.

## 4 Organelle dysfunction and metabolic remodeling in senescent cardiomyocytes

Cellular dysfunction in aging cardiomyocytes is closely tied to progressive mitochondrial deterioration and metabolic remodeling. Proton leak increases in the mitochondria of cardiomyocytes isolated from the hearts of aged mice and rats [48]. In human induced pluripotent stem cell (iPSC)-derived cardiomyocytes (hiPSC CMs), doxorubicin (DOX), a widely used chemotherapeutic agent frequently employed to model cardiomyocyte senescence, increases SA-β-Gal activity, DNA damage, and p21 transcripts [49], [50], with the inner mitochondrial membrane (IMM) exhibiting alterations in its morphology and dynamics that disrupt oxidative phosphorylation (OXPHOS) [49]. In the human cardiomyocyte cell line AC16, DOX enhances glycolysis and reprograms multiple metabolic pathways, including the tricarboxylic acid (TCA) cycle, fatty acid β-oxidation, glycerolipid metabolism, and the carnitine shuttle [51]. Of note, the aged heart exhibits features of pseudohypoxia associated with epigenetic dysregulation. Cardiomyocytes isolated from aged hearts display increased H3K27 acetylation, as well as reduced H3K4 monomethylation and H3K27 trimethylation, changes associated with elevated expression of the transcriptional coactivator p300, and concomitant suppression of OXPHOS gene expression [52]; hypoxia enhances glycolysis-mediated ATP production in atrial HL-1 cells, which is inhibited by suppression of p300/CBP [52]. Indeed, pharmacological inhibition of p300/CBP has been shown to ameliorate cardiac systolic dysfunction in aged mice, with isolated cardiomyocytes also demonstrating improved contractility accompanied by a reduction in Hk2 gene expression [52]. Alterations in mitochondrial redox homeostasis further contribute to cardiomyocyte dysfunction. Manganese superoxide dismutase (MnSOD) is a mitochondrial matrix enzyme that detoxifies superoxide radicals into hydrogen peroxide, thereby protecting mitochondria from oxidative damage and maintaining redox homeostasis. However, constitutive activation of MnSOD increases SA-β-gal activity, and proteins p53 and p21 levels in the heart, while reducing mitochondrial size in cardiomyocytes, and has been shown to induce a dilated cardiomyopathy-like phenotype in experimental models [53]. At the functional level, these alterations are associated with electrical remodeling and increased arrhythmogenic risk in the aging heart [54]. Application of DOX to hiPSC CMs prolongs multicellular QTc and single-cell action potential duration (APD), with an increased incidence of APD variability and delayed afterdepolarizations (DADs) [50], which occur in association with augmentation of the late sodium current (INaL) and reduction in the delayed rectifier potassium current (IKr) [50]. Moreover, sarcoplasmic reticulum (SR) Ca^2+^ content is reduced due to SERCA2 downregulation and enhanced RyR2-mediated Ca^2+^ leak, with these electrical and intracellular Ca^2+^ alterations largely attributable to increased CaMKII activity in DOX-treated cardiomyocytes, while cardiomyocytes extracted from ventricles exhibit prolonged APD, enhanced INaL, and increased CaMKII phosphorylation [50]. Altogether, these findings suggest that mitochondrial dysfunction, metabolic reprogramming, redox imbalance, and electrophysiological remodeling contribute to cardiomyocyte senescence and aging-associated cardiac dysfunction.

## 5 Downstream destabilization of cardiomyocyte homeostasis: autophagy loss, telomere dysfunction, inflammation, and environmental stressors

Aging is associated with a decline in autophagic response in systemic organs, including the heart. The GSK-3β/serine/threonine-protein kinase ULK1 (Ulk1) axis plays a crucial role in the regulation of cardiac autophagy [26], as GSK-3β phosphorylates Ulk1 to stimulate autophagy. Indeed, cardiomyocyte-specific expression of a non-phosphorylatable ULK1 mutant results in cardiac dysfunction, hypertrophy, and fibrosis, along with increased levels of p53 and p16, and DNA damage in murine hearts at 7 months [26]. Spermidine enhances autophagy flux in cardiomyocytes of both young and aged mice, ameliorates left ventricle (LV) diastolic dysfunction in aged mice, and has been shown to extend lifespan in experimental models [55], with this cardioprotective effect shown to be lost in Atg5 knockout mice, supporting a key role of autophagy in mediating the cardioprotective effect of spermidine [55]. Together, these findings highlight impaired autophagy as a key mechanism of cardiomyocyte aging and support autophagy restoration as a potential therapeutic strategy for cardiac aging.

Telomere attrition develops in the aged heart as well as in patients with heart failure [31], [47], [56], [57], with telomerase (TERT) inhibition suppressing the differentiation toward cardiomyocytes in hiPSC. hiPSC-CMs associated with short telomeres exhibit diminished oxygen consumption rate (OCR), a reduction in beat rates, increased p21 levels, DNA damage, and SA-β-Gal activity, and show increased susceptibility to mitochondrial dysfunction and cell death [57], [58]. NFκB signaling is upregulated in the aged heart, and its cardiomyocyte-specific activation enhances cardiac fibrosis, infiltration of myocardial CD45-, CD11b-, and F4/80-positive cells, and increases *Emr1*, *Icam1*, *Ccl2*, and Cxcl10 transcripts in mice [59].

In addition to these intrinsic processes, extrinsic factors have also been suggested to contribute to aging in the heart and cardiomyocytes. Environmental particulate pollutants, including particulate matter with an aerodynamic diameter of ≤2.5 μm (PM2.5) and nano-plastics (NPs), are increasingly recognized as modulators of cardiovascular aging. NP exposure has been shown to impair LV systolic function, increase cardiac fibrosis, and elevate ROS levels, accompanied by increased levels of senescence- and inflammation-associated proteins (p53, p21, p16, TNF-α, IL-1β, and IL-6) in the mouse heart [60]. In vitro, NPs are internalized by cardiomyocytes and induce similar senescence and inflammatory responses [60]. In the HFpEF model, concentrated ambient PM2.5 (CAP) increases cardiac fibrosis and reduces LV systolic and diastolic function [61]. In vitro studies further demonstrate that CAP increases ROS and DNA damage and promotes cell death or senescence in cardiomyocytes [61], [62]. Together, these studies highlight that cardiomyocyte aging emerges from multilayered intrinsic and environmental stress responses that converge on the progressive destabilization of myocardial homeostasis.

In addition to cardiomyocyte-dependent mechanisms, age-associated ventricular remodeling is influenced by dysfunction of non-cardiomyocyte populations, as discussed in subsequent sections.

## 6 Endothelial cells (ECs) in the aging ventricle

Endothelial senescence has emerged as an important mechanism contributing to age-associated ventricular remodeling. Aging is associated with induction of transcriptional dysregulation in cardiac endothelial cells, including alterations in up to 700 genes and a reduction in the Apelin receptor-enriched subtype, thus highlighting distinct age-related molecular and cellular changes in the heart endothelium [63]. The aged heart exhibits a reduction in miR-145 levels, resulting in increased levels of semaphorin-3A in cardiac endothelial cells (ECs), which is associated with reduced ventricular nerve density [21]. Single-cell dual-omics analyses further reveal substantial cell type-specific and intra-cell type heterogeneity among cardiac non-cardiomyocytes in the aged heart [64]. Although aging-associated transcriptomic changes appear less pronounced in ECs than in macrophages, which exhibit the strongest response to aging, endothelial dysfunction may nevertheless substantially influence myocardial homeostasis through altered vascular integrity, neurovascular signaling, and intercellular communication [64]. Collectively, these findings support the concept that cardiac aging emerges through multicellular and cell type-specific remodeling processes involving both endothelial and other non-cardiomyocyte populations. Aging-related alterations in additional non-cardiomyocyte populations are discussed in the following section.

## 7 Fibroblasts in the aging ventricle

Cardiac fibroblasts undergo profound age-associated remodeling that drives extracellular matrix (ECM) remodeling and alters the myocardial microenvironment. However, these changes are not simply driven by increased fibroblast proliferation or transcriptional activation of canonical ECM genes. Instead, accumulating evidence suggests that fibroblast senescence functions as an important regulator of cardiac aging by amplifying inflammatory signaling, promoting anti-angiogenic and osteogenic gene signatures, and compromising reparative capacity [65]. Cardiac fibroblasts exhibit high proliferative and transcriptional activity during the neonatal period, with proliferation rates exceeding 50% at postnatal day 1, rapidly declining to approximately 10% by 4 weeks of age and further decreasing to ~2% in early adulthood, where they remain low throughout life. Notably, aging is associated with relatively limited changes in fibroblast proliferation, collagen/ECM-related gene expression, or DNA methylation, suggesting that age-associated cardiac fibrosis may not primarily result from intrinsic fibroblast proliferative expansion or major collagen-related transcriptional reprogramming [66]. An expanded population of osteogenic fibroblasts emerges in the epicardium of aged hearts, characterized by increased transcript levels of Runx2, the master transcription factor regulating osteogenic differentiation, in isolated cardiac fibroblasts [65]. While GATA-binding protein 4 (GATA4) plays roles in cardiomyocyte proliferation, differentiation, survival, and hypertrophy, its role in fibroblasts has remained unclear. However, fibroblast-specific deletion of GATA4 in Tcf21-lineage cells increases p16 expression and SA-β-gal activity in the heart, whereas cardiomyocyte-specific depletion does not produce this phenotype, suggesting that GATA4 may function as a fibroblast-intrinsic regulator of myocardial aging [67]. Senescence also reduces the efficiency of direct cardiac reprogramming, as senescent fibroblasts are less capable of being converted into cardiomyocytes. Recent studies identify the nuclear receptor Nr4a3 as an important mediator of this barrier; notably, Nr4a3 depletion reverses senescence-associated changes, restores a regenerative chromatin state, and improves cardiac function after myocardial infarction in preclinical models [68]. Mechanical cues within the cardiac ECM regulate fibroblast fate, with low-stiffness substrates suppressing myofibroblast activation while promoting a premature senescence program enriched for SASP genes, highlighting the sensitivity of fibroblasts to age-related ECM remodeling [69]. Cardiac fibroblasts are reported to be regulated through organ-organ interactions. Notably, visceral adipose tissue-derived osteopontin increases in circulation with aging, and promotes myocardial fibrosis by suppressing cardiac fibroblast senescence, resulting in enhanced myofibroblast activation and ECM production, thereby contributing to age-related cardiac dysfunction [70]. Another example of organ-to-heart communication involves brown adipose tissue (BAT)-derived procollagen C-endopeptidase enhancer-1 (PCPE-1), a secreted profibrotic factor whose expression increases with aging and obesity [71], [72]. PCPE-1 promotes procollagen processing and fibrillogenesis, and its circulating levels are elevated in aged mice. Systemic depletion of PCPE-1 was shown to ameliorate LV fibrosis and diastolic dysfunction in aged HFpEF models, whereas BAT-specific overexpression aggravates these phenotypes under dietary obesity, supporting a role for the BAT-heart axis in age-associated myocardial fibrosis and HFpEF [71]. Together, these observations support a model in which fibroblast senescence contributes to age-related LV remodeling through coordinated ECM remodeling, fibroblast-intrinsic signaling, immune interactions, and systemic metabolic-inflammatory inputs.

## 8 Immune cells in the aging ventricle

Immune-cell senescence has emerged as an important determinant of ventricular aging, reshaping the inflammatory and reparative landscape that governs myocardial homeostasis [16]. With aging, CD8^+^ T cells increase in the heart, while, in contrast, CD4^+^ T cells decline in immunodeficient mice, suggesting that T cells contribute to age-related myocardial inflammation and functional decline [16]. However, these observations derive primarily from experimental models, and the extent to which similar dynamics occur in human cardiac aging remains to be fully defined. Single-cell dual-omics profiling of the aged heart demonstrates that immune and stromal populations undergo profound remodeling, with macrophages, neutrophils, and selected fibroblast-associated clusters exhibiting the most pronounced transcriptional and epigenetic reprogramming [64]. Spatial and computational analyses of the aging mouse heart reveal enrichment of a profibrotic macrophage phenotype within large vessel niches, where tissue-resident macrophages (CD68^+^Lyve1^+^) colocalize with fibroblasts, and bone marrow-derived macrophages (CD68^+^Lyve1^−^) associate with B cells. Cell-cell communication modeling suggests activation of the C3-C3ar1 signaling axis between C3-expressing fibroblasts and C3ar1-expressing macrophages, both of which are increased with aging, highlighting macrophage-associated inflammatory remodeling within the vascular niche [35]. Interestingly, senolysis was shown to reduce Lyve1^−^ macrophages, indicating a functional link between senescence and immune remodeling [35].

These shifts include the emergence of senescent and SASP-producing immune subsets that remodel intercellular communication networks, supporting a role for immune senescence as a potential organizer of the aged cardiac microenvironment.

The aged murine heart exhibits elevated mitochondrial Arginase 2 (Arg2) in non-cardiomyocytes, including cardiac macrophages, fibroblasts, and endothelial cells [73]. Arg2 is associated with increased IL-1β production in macrophages, thereby promoting cardiomyocyte apoptosis, matrix production in fibroblasts, and endothelial-mesenchymal transition (EndMT) in ECs [73], illustrating how age-associated metabolic and inflammatory signaling in non-cardiomyocyte populations can coordinately drive multicellular remodeling within the aging myocardium.

Systemic aging also influences immune populations trafficking into or interacting with the LV. Peripheral blood mononuclear cells from older adults display transcriptomic enrichment of DNA damage, i.e., telomere stress-induced senescence and the nicotinamide adenine dinucleotide (NAD+) signaling pathway, as well as oxidative stress-induced senescence [74]. Remarkably, these signatures are acutely modifiable with a single bout of high-intensity exercise, demonstrating the dynamic plasticity of immune cells even later in life [74]. Given the continuous exchange between circulating leukocytes and cardiac tissues, particularly during homeostatic surveillance or injury, these systemic senescence programs are likely to influence immune behavior within the aging LV.

Collectively, these findings depict immune-cell senescence as a multi-level process affecting both cardiac-resident and circulating leukocyte populations, integrating the altered cellular composition, metabolic stress responses, SASP activity, and transcriptomic remodeling. These mechanisms contribute to the chronic inflammatory milieu characteristic of LV aging and may help identify potential targets for modulating immune-associated cardiac decline.

While immune-cell senescence contributes substantially to aging-associated remodeling in the ventricle, aging of the atrium involves distinct structural, cellular, and inflammatory programs that promote the formation of a unique arrhythmogenic substrate.

## 9 Characterization of the aged atrium

Beyond physiological aging, metabolic and systemic inflammatory disorders, including obesity, diabetes, and chronic inflammatory diseases, have emerged as major accelerants of atrial remodeling. These conditions are associated with the development of atrial myopathy characterized by atrial enlargement, fibrosis, and impaired compliance, largely mediated by epicardial adipose tissue-derived inflammatory signaling, and are associated with an increased risk of stroke that exceeds conventional thromboembolic risk stratification [75]. As in the ventricle, these age-related alterations are further exacerbated by cardiometabolic comorbidities, contributing to both structural and functional abnormalities of the atrium. At the molecular level, aging is associated with increased levels of senescence markers, including p53 and p21, in LAAs [76]. This senescence signature is further amplified in atrial fibrillation (AF), with higher levels of p53, p21, and p16 consistently observed in atrial tissues from patients with AF compared with those in sinus rhythm [76], [77]. Notably, AF progression in human atrial tissue is accompanied by a stepwise accumulation of senescence markers, particularly p53 and p16, together with enhanced thrombogenic signaling and endothelial dysfunction, suggesting that cellular senescence may contribute to atrial remodeling and prothrombotic risk rather than merely reflecting tissue aging [77]. While these observations are supported by human tissue analyses, mechanistic insight into causality remains largely derived from experimental systems.

Experimental models further support a contributory role for senescence in atrial dysfunction. In rat models of aging and myocardial infarction (MI), senescent cell accumulation within the atrium is associated with fibrosis, conduction delay, and increased AF susceptibility, whereas senolytic interventions attenuate these alterations, supporting the potential of targeting cellular senescence in atrial remodeling [78]. At the tissue level, fibrotic remodeling creates a distinct arrhythmogenic substrate; studies in a goat model demonstrate that fibrotic atrial regions exhibit slowed conduction and unidirectional propagation, providing a mechanistic basis for AF initiation and maintenance [79]. Consistent with these findings, analyses of human atrial appendages indicate that aging itself is an independent determinant of atrial fibrosis, associated with enhanced profibrotic signaling (notably TGF-β), reduced hypoxia-responsive and angiogenic pathways, and an imbalance in MMP/TIMP activity favoring extracellular matrix accumulation [80].

Collectively, these findings suggest that atrial aging is a multifaceted process involving both adaptive and maladaptive responses, including inflammation-associated remodeling, cell type-specific senescence, and fibrosis-mediated electrical disorganization, consistent with a context-dependent role of senescence. These alterations converge to create a vulnerable substrate that promotes the initiation and maintenance of AF, distinct from, yet conceptually linked to, ventricular aging biology.

## 10 Atrial cardiomyocyte senescence in AF and aging

Accumulating evidence indicates that atrial cardiomyocyte senescence is not merely a consequence of chronological aging but is also associated with AF-related stress, potentially contributing to maladaptive atrial remodeling. Both human and experimental studies demonstrate that AF itself has been shown to be associated with, and may promote a senescent phenotype in atrial cardiomyocytes, characterized by upregulation of p16 and p21, increased DNA damage, and elevated *Cdkn1a* (p21) transcripts in the left atrial appendage, correlating with AF recurrence [81]. Mechanistically, oxidative DNA damage is increasingly recognized as an important upstream contributor to atrial cardiomyocyte senescence and dysfunction. In this context, excessive activation of poly(ADP-ribose) polymerase 1 (PARP1) in response to DNA damage is associated with depletion of intracellular NAD^+^ pools, amplification of DNA damage, and impaired contractile function in HL-1 atrial cardiomyocytes. Importantly, pharmacological inhibition of PARP1 or NAD^+^ replenishment preserves cardiomyocyte function, supporting the PARP1–NAD^+^ axis as a key regulator of atrial cardiomyocyte vulnerability in AF [82].

Beyond AF-related stress, age-associated senescence programs initially described in ventricular cardiomyocytes appear to extend to the atrium. For example, prominin-2, a cardiomyocyte-specific senescence marker identified in the aging ventricle, is similarly upregulated in atrial tissue from patients with heart failure with preserved ejection fraction (HFpEF), suggesting conserved cardiomyocyte senescence pathways across cardiac regions [41]. Metabolic stress further accelerates this process, as advanced glycation end-products have been shown to induce atrial cardiomyocyte senescence via the RAGE–p16/Rb pathway, promoting electrical remodeling and increasing AF susceptibility in diabetic settings [83].

Collectively, these findings support a model in which atrial cardiomyocyte senescence may represent a central, cell-autonomous process that integrates aging, metabolic stress, and arrhythmic burden, and may contribute to atrial dysfunction.

## 11 Atrial EC senescence in AF and aging

Aging is associated with capillary rarefaction in the LV [21], [84], and similar alterations appear to occur in the atrium. Indeed, reduced capillary density has been reported in atrial tissue from patients with AF, an effect partially ameliorated by ACE inhibitor therapy [85], suggesting that microvascular dysfunction may contribute to atrial remodeling. In parallel, AF is characterized by a hypercoagulable state that promotes thrombus formation and thromboembolic events. Emerging evidence indicates that coagulation factors directly modulate endothelial cell (EC) function and senescence. In porcine atrial ECs, activated coagulation factor Xa increases the expression of p53, p21, TGF-β, and collagen 3a via the angiotensin II type 1 receptor (AT1R)/NADPH oxidase/sodium–glucose cotransporter (SGLT1/2) signaling axis, suggesting a mechanistic link between coagulation signaling and atrial EC senescence [86]. Similarly, thrombin induces p53, p21, and p16 expression in atrial ECs, further supporting a role for coagulation-associated endothelial senescence [87]. In addition to these pro-senescent signals, atrial endothelial dysfunction is associated with structural remodeling through endothelial-to-mesenchymal transition (EndMT). Atrial endocardial endothelial cells (AEECs) undergo TGF-β–mediated EndMT via the miR-181b/semaphorin-3A/LIMK/cofilin axis, promoting endocardial fibrosis in the atrium [88].

Collectively, these findings suggest that atrial endothelial senescence and dysfunction contribute to microvascular rarefaction and coagulation-related signaling, thereby promoting fibrotic remodeling and AF progression.

## 12 Atrial fibroblast senescence in AF and aging

Left atrial tissues from aged mice and patients with AF consistently exhibit increased fibrosis [76], [78], [85], [89]. This fibrotic remodeling is accompanied by increased levels of p53, p21, Col1A1, and p300 in the atrium with aging [76]. In vitro studies show that replicative senescent human atrial fibroblasts exhibit elevated levels of these proteins as well as an increase in SA-β-gal activity [76]. Mechanistically, the acetyltransferase p300 emerges as a key regulator of fibroblast senescence. Inhibition of p300 reduces SA-β-gal activity and decreases the expression of p53, p21, and Col1A1 in senescent atrial fibroblasts, with similar effects observed in the atrium of aged CAG-Cre; p300^flox/+^ mice [76]. Consistent with this, hypertension-associated mechanical stress activates the p300/p53/Smad3 pathway in atrial fibroblasts, suggesting that hemodynamic load may promote a pro-senescent and pro-fibrotic phenotype [90]. In contrast, epigenetic regulators such as enhancer of zeste homolog 2 (Ezh2) act as a brake on fibroblast senescence. Ezh2 suppresses replicative senescence in atrial fibroblasts through H3K27me3-mediated repression of p16 and tissue inhibitor of metalloproteinases 4 (Timp4), and is associated with attenuation of age-related atrial fibrosis and the development of an arrhythmogenic substrate for AF. Notably, Ezh2 expression declines with aging in the left atrium and in isolated atrial fibroblasts, suggesting a progressive loss of this protective mechanism [91].

Collectively, these findings position atrial fibroblast senescence as a dynamic and regulatable process integrating aging, mechanical stress, and epigenetic control, thereby driving atrial fibrosis and electrical vulnerability. The balance between pro-senescent and anti-senescent pathways may critically determine the extent of fibrotic remodeling and AF susceptibility, highlighting fibroblast senescence as a potential therapeutic target for modifying the structural substrate of AF.

## 13 Cellular senescence in atrial immune cells

In addition to structural cell senescence, immune cell senescence has recently emerged as a novel modulator of atrial electrophysiology. Senescent CD8^+^ T cells are selectively expanded in patients with AF and have been reported to exert proarrhythmic effects by releasing interferon-γ, which has been shown to disrupt cardiomyocyte calcium handling, thereby promoting electrical instability [92]. These findings highlight the role of immune senescence as a non-cell-autonomous contributor to atrial arrhythmogenesis and suggest that senescent immune cell signatures may serve as both biomarkers and therapeutic targets in AF.

## 14 Biomarkers

### 14.1 Biomarkers of senescence and biological aging in the ventricle

As outlined in the preceding sections, aged ventricles undergo coordinated biological alterations characterized by genomic instability, cellular senescence, mitochondrial dysfunction, metabolic derangement, neurovascular dysregulation, and inflammatory activation. In this section, attention is focused on highlighting key biomarkers associated with these interconnected processes, while acknowledging that many of these markers are not specific to cardiac tissue and may reflect systemic aging or inflammation rather than myocardial senescence. In all original studies discussed here, different terms were used to describe the tissue sources used, which include “LV,” “heart,” “myocardium,” and “cardiac.” While these descriptions generally refer to left ventricular tissue, the terminology as used in each respective publication is retained to avoid ambiguity. Aged LVs exhibit elevated transcript levels of *Cdkn1a*, *Cdkn2a*, *Il1b*, *Mmp9*, *Ifng*, and *Cxcl13* [16], [41], [46]; aged hearts show an increase in the cross-sectional area (CSA) of cardiomyocytes, enhanced fibrosis, SA-β-Gal activity, and high protein levels of p53, p21, p16, IL1β, IL6, and CaMKII [44], [47]; and IGFBP7 increases in the murine aged heart and is associated with increased cardiac senescence through the activation of IGF-1R/IRS/AKT signaling-dependent FOXO3a suppression, inhibiting DNA repair and ROS detoxification [6].

Telomere attrition provides a unifying mechanistic biomarker. Telomere shortening develops in the LVs with aging and heart disease and is negatively correlated with heart weight [56]. In addition to findings from human studies [56], experimental investigations in mice likewise demonstrate telomere attrition in the aging heart [31], [47]. Another study demonstrates that postmitotic cardiomyocytes accumulate telomere-associated DNA damage foci independently of telomere length, producing a pro-fibrotic and hypertrophic SASP; genetic or pharmacological senolysis reverses hypertrophy and fibrosis in the aging myocardium, suggesting potential translational relevance of these biomarkers [40].

Structural and microenvironmental biomarkers further refine our understanding of ventricular aging. MMP-9, implicated in ECM turnover, increases with aging, contributing to the inflammatory and angiogenic state of the aging ventricle, with its deletion enhancing microvascular preservation and reduces vascular permeability, thus providing a mechanistic foothold for early detection of pathological remodeling [93], [94].

A newly emerging cardiomyocyte-intrinsic senescence marker is Prom2, which increases in aged murine ventricles and cardiomyocytes purified from the LVs [41]. Prom2 induction follows p53-dependent telomere and genotoxic stress, and is sufficient to trigger H9c2 cardiomyocyte hypertrophy, senescence, and cytotoxic-stress resistance, with its suppression attenuating DOX-induced senescence, thus suggesting Prom2 as a candidate biomarker and effector of ventricular aging [41].

Neurovascular signaling represents an additional layer of ventricle-specific biomarkers. Aged hearts and ECs isolated from aged hearts exhibit elevated Sema3A levels, while age-related loss of endothelial miR-145 derepresses Sema3A, producing sympathetic denervation and electrical instability in the aged ventricle, while senolytic clearance restores axonal density and normalizes neurovascular signaling, thus highlighting Sema3A as an actionable biomarker of ventricular senescence [21]. Key molecular and structural features associated with ventricular aging are summarized in Table 1, Table 2, Table 3, and the Figure; however, these findings have been derived from diverse experimental systems and species, including mice, rats, and humans, and therefore require further validation to establish their translational relevance, specificity, and clinical utility.

**Table 1:** Aging-related markers and experimental conditions in human and rat hearts and cardiomyocytes.

Experimental subjects	Aging-related marker	Organ/cells studied	Reference
Humans			
< 40- vs. > 40-year-old males	Reduction in inferior-wall MIBG uptake	Heart	PMID: 7769454
From 0 to 104 years old	Telomere attrition	LV	PMID: 23929129
< 40 vs. > 40 years old	Protein p53	Myocardium	PMID: 33754023
40 vs. > 40 years old	mtDNA deletion	Cardiac muscle	PMID: 26632292
From 19 to 73 years old	Reduction in cardiomyocyte renewal	Isolated cardiomyocytes from the LV	PMID: 19342590
From 0.4 to 82 years old	Somatic mutations, polyploidization	Isolated cardiomyocytes from the LV	PMID: 36051457
Rats			
6 vs. 27 months old: F344BNF1 male rats	Mitochondrial DNA damage and loss	Heart	PMID: 29969715
3 vs. 25 months old: F344BNF1 male rats	Suppression of proteostasis	Heart	PMID: 18533226
2.5 vs. months old: Wister rats	Reduction in norepinephrine transporter function (PET study)	Heart	PMID: 30042495
1, 4 vs. 10, 20 months old: Sprague-Dawley rats	Apoptosis (TUNEL, cleaved caspase-3), increased myocyte CSA and extramyocyte space, diminished myocyte number	LV	PMID: 32899456
5 vs. 24 months old: Sprague-Dawley rats	Oxidative DNA damage (8-OHdG)	Myocardium	PMID: 25384832
5–7 vs. 25–30 months old: Fischer 344 rats	Mitochondrial proton leak	Isolated cardiomyocytes from the heart	PMID: 33319746

CSA, cross-sectional area; DNA, deoxyribonucleic acid; F344BNF1, Fischer 344 Brown Norway; LV, left ventricle; MIBG, 123I-metaiodobenzylguanidine; TUNEL, terminal deoxynucleotidyl transferase dUTP nick end labeling.

**Table 2:** Aging-related markers and experimental conditions in murine hearts and cardiomyocytes.

Experimental subjects	Aging-related markers	Organ/cells studied	Reference
4- vs. 24-month-old male mice: C57BL/6	Telomere attrition, mitochondrial DNA damage, reduced NAD^+^, increased 8-oxo-dG	Heart	PMID: 33934090
2–3 vs. 18 months old: C57BL/6N	Telomere attrition, DNA damage, fibrosis, proteins p53 and p21, reduction in miR-30a-5p transcript	Heart	PMID: 39511427
8 vs. 69–73 months old: C57BL/6	Reduction in IκBa, increase in nuclear ReIA (proteins), and *Cdkn2a* and *II6* transcripts	Heart	PMID: 39011799
3 vs. 24 months old: C57BL/6	Reduction in protein PCSK6 and increase in proteins p16, p21, and DDIT3	Heart	PMID: 35456517
3 vs. 24 months old: C57BL/6	Increased cardiomyocyte CSA, myocardial fibrosis, SA-β-Gal activity, NFκB signaling, proteins p53, p16, IL-1β, IL-6, and CaMKII	Heart	PMID: 36462537
3 vs. 18 months old: C57BL/6	p53, p21 (transcripts and proteins), lipofuscin, DNA damage, increase in myocyte CSA, HW/BW ratio, fibrosis, apoptosis (TUNEL)	Heart	PMID: 39857807
4 vs. 24 months old: C57BL/6	Suppressed autophagy	Heart	PMID: 32627317
6 vs. 18 months old: C57BL/6	Increases in miR-34a and reduction in miR-145	Heart	PMID: 23426265, 37616346
3 vs. 24 months old: C57BL/6	Protein IGFBP7	Heart	PMID: 39196168
3 vs. 18 months old: C57BL/6JRj	LV larger vessel-associated cellular niches as hotspots in inflammation and cardiac aging	LV	PMID: 41090219
2 vs. 22 months old	Increased cardiomyocyte CSA, myocardial fibrosis, transcripts *Cdkn1a*, *Cdkn2a*, and *Il1b*, reduction in MLF1 (transcript and protein)	LV	PMID: 39657728
3 vs. > 16 and >22 months old: C57BL/6J	Capillary rarefaction at >22 months old, reduction in axon density at >16 months old	LV	PMID: 37616346
63–70 vs. ≈ 650 days old	Capillary rarefaction	LV	PMID: 7954608
6–9 vs. 15–18 months old: C57BL/6J	*MMP9* transcript, vascular permeability	LV	PMID: 24658018
6–9, 12–15, 18–24, and 26–34 months old: C57BL/6J	Age-dependent increase in M1 macrophage, reduction in M2 macrophage	LV	PMID: 25883218
6 vs. 24 months old	Phosphorylation of GSK-3β (indicating inactivation)	LV	PMID: 34778891
2–3 vs. 12–15 months old: C57BL/6J	LV systolic dysfunction, myocardial fibrosis, cardiomyocyte hypertrophy, inflammatory gene expression: *Tnf*, *Ifng*, *Cxcl13*, and *Ccl2*, accumulation of activated CD4^+^ Foxp3-IFN-γ cells in heart-draining lymph nodes, reduction in CD4^+^/CD8^+^ T cell ratio	Myocardium	PMID: 28255084
4 vs. 24 months old: C57BL/6	Proteins p53, p21 and p16	Myocardium	PMID: 31543347
2–10 vs. > 15 months old: C57BL/6NNia	DNA damage	Myocardium	PMID: 3820319
3 vs. > 12 months old: C57BL/6N	Protein p53	Myocardium	PMID: 33754023
2 vs. 15 months old	p21 (transcript and protein), fibrosis	Myocardium	PMID: 34162223
3 vs. 24 months old: C57BL/6	Protein phosphor-NFκB, transcripts *Tnfa, Il1b, Il6,* and *Il10*	Myocardium	PMID: 37958823
3 vs. 24 months old	NAD^+^ reduction	Myocardium	PMID: 38098220
2–3 vs. 12–15 months old: C57BL/6J	Increase in CD8+ T cells, reduction in CD4+ T cells, increase in transcripts *Tnf, Ccl2, Cxcl1, Ifng, Ccl5*, and *Cxcl13*	Myocardium	PMID: 28255084
3 vs. 20 months old: C57BL/6J	Transcripts *Cdkn1a* (p21) and *Cdkn2a* (p16), Prom2 (protein and transcript), increase in LV mass and IVSTd	LV and isolated LV cardiomyocytes	PMID: 38757782
2 vs. 18 months old: C57BL/6J	Pseudohypoxia, reduction in OXPHOS genes and NAD^+^	Isolated LV cardiomyocytes	PMID: 37681309
3 vs. 20 months old: C57BL/6J	Transcript *Prom2*	Isolated LV cardiomyocytes	PMID: 38757782
2 vs. 18 months old: C57BL/6J	Epigenetic dysregulation, increase in transcriptional coactivator p300, reduction in OXPHOS genes	Isolated LV cardiomyocytes	PMID: 37681309
7 weeks vs. 18 months old	Prolonged APD, enhanced lNal, increased AAMKII phosphorylation	Isolated ventricular cardiomyocytes	PMID: 35836799
4–6 vs. 24–26 months old: C57BL/6	Mitochondrial proton leak	Isolated cardiomyocytes	PMID: 33319746
3 vs. 15 and 30 months old: C57BL/6	TAF, protein p21	Cardiomyocytes isolated from the myocardium	PMID: 30737259
3 vs. 20 and 24 months old: C57BL/6	Transcripts *Cdkn1a* (p21) and *Cdkn2a* (p16), SA-β-Gal activity	Cardiomyocytes isolated from the myocardium	PMID: 30737259

AAMKII, Ca2+/calmodulin-dependent protein kinase II; APD, action potential duration; CSA, cross-sectional area; DNA, deoxyribonucleic acid; HW/BW ratio, heat weight/body weight ratio; lNal, late sodium current; IVSTd, interventricular septal end-diastolic thickness; LV, left ventricle; miR, microRNA; NAD+, nicotinamide adenine dinucleotide; OXPHOS, oxidative phosphorylation; SA–Gal, senescence-associated β-galactosidase; TUNEL, terminal deoxynucleotidyl transferase dUTP nick end labeling.

**Table 3:** Aging-related markers and experimental conditions in non-cardiomyocytes of the murine heart.

Experimental subjects	Aging-related markers	Organ/cells studied	Reference
3 vs. 20 months old: C57BL/6J	Semaphorin-3A, SA-β-Gal activity	Cardiac endothelial cells (Sema3A), Heart (SA-β-Gal activity)	PMID: 37616346
12 weeks vs. > 18 months old: C57BL/6	Increase in osteogenic fibroblasts in the epicardium, transcript *Runx2* in isolated cardiac fibroblasts	Cardiac fibroblasts (Runx2), epicardium (osteogenic fibroblasts)	PMID: 31723062
Postnatal days 1, 4, 14, 24 weeks and 1 year old: (C57BL/6	High proliferation at birth (postnatal day 1) (> 50%), declines by up to 10% by 4 weeks of age, further decreases by up to 2% in early adulthood	Cardiac fibroblasts	PMID: 33180747
8 weeks vs. 18 and 30 months old: C57BL/6	Increase in neutrophils and macrophages in the heart but no change in monocytes	Cardiac neutrophils and macrophages	PMID: 29339450
3–4 vs. 20–22 months old	Increase in Arg2 in non-cardiomyocytes	Cardiac macrophages, fibroblasts, and endothelial cells	PMID: 41187268

SA–Gal, senescence-associated β-galactosidase.

### 14.2 Biomarkers of senescence in the atrium: a unifying framework of molecular, metabolic, structural, and immune aging

A growing body of evidence suggests that the atrium may function as a sensitive biosensor of cardiovascular aging, where inflammation, extracellular matrix remodeling, fibrosis, mitochondrial dysfunction, metabolic decline, electrophysiological vulnerability, and cellular senescence act in concert to produce a syndrome increasingly conceptualized as atrial myopathy [76], [78], [80], [89], [91], [95], although many of these features are not specific to the atrium and may reflect systemic aging processes. This framework captures the shared biological substrate underlying AF, HFpEF, ischemic stroke, and even cognitive decline and has spurred efforts to define atrial senescence through multimodal biomarkers including circulating molecules, tissue-based signatures, imaging metrics, and immune-cell phenotypes [96]. Across human cohorts, cardiovascular multimorbidity parallels an acceleration of atrial biological age, characterized by DNA damage and epigenetic changes, impaired mitochondrial energetics, innate immune activation, and cellular senescence, which together may render the aged atrium more susceptible to metabolic and hemodynamic stressors [97]. Impaired left atrial strain in older adults correlates with vascular cognitive impairment, cerebral small-vessel disease burden, and circulating inflammatory and neuronal-injury markers, suggesting that mechanical atrial dysfunction may represent an upstream biomarker linking atrial myopathy to cerebrovascular aging [98]. Speckle-tracking echocardiography in murine models demonstrates that aging, angiotensin II infusion, and heart failure with preserved ejection fraction impair left atrial reservoir, conduit, and contractile function and increase AF inducibility, thus providing a platform for mechanistic and therapeutic studies of atrial myopathy [99]. Studies of the atrium in aged humans and mice show that proteins p53, p21, p16, Col—I, Col-III, Vimentin, as well as SA-β-Gal activity, increase with aging [76], [91]. Human cardiac stem/progenitor cells (hCPC) isolated from aged atrial appendages also exhibit high SA-β-Gal activity and *Cdkn2a* (p16) transcripts [95]. Metabolic profiling highlights β-hydroxybutyrate and citrate synthase activity as markers of mitochondrial integrity, with their reduction in the left atrium occurring following atrial structural remodeling and in low-voltage substrate in older adults with AF [100]. Interestingly, aged AF patients exhibit elevated levels of apoptosis, ROS, fibrosis, proteins TGF-β1, Col I, Col III, Drp-1, Fis-1, as well as a reduction in proteins Pgc-1α, Mfn1, Mfn2, Opa1, mitochondrial complex proteins (I-V) in the left atrium compared to non-AF patients [100], suggesting extensive mitochondrial and metabolic remodeling. Transcriptomic analyses further reinforce the systemic nature of atrial aging, identifying a 12-gene signature (*CAST*, *ASAH1*, *MAFB*, *VCAN*, *DDIT4*, *FTL*, *HEXB*, *PROS1*, *BNIP3L*, *PABPC1*, *YBX3*, and *S100A6*), including lysosome-associated genes, such as ferritin light chain (FTL) and acid ceramidase (ASAH1), which are consistently upregulated in both blood and left atrial tissue from individuals with AF compared to those with sinus rhythm (SR), indicating that lysosome-related genes may be involved in AF pathophysiology [101].

Inflammation and immune aging are closely interrelated with atrial vulnerability. Expansion of senescent CD8^+^ T cells in peripheral blood and atrial tissue predicts AF prevalence and postprocedural AF recurrence [92]. Their heightened secretion of interferon-γ is associated with proarrhythmic calcium-handling abnormalities in human atrial cardiomyocytes, suggesting that immune senescence may represent an electrophysiologically active contributor to AF pathogenesis, although further validation is required [92].

Altogether, current evidence indicates the atrium as a key biosensor of cardiovascular aging, in which molecular senescence, mitochondrial and metabolic dysfunction, structural remodeling, electrophysiological vulnerability, and immune activation converge to form the substrate of atrial myopathy. The integration of multimodal biomarkers, i.e., tissue signatures, circulating factors, imaging indices, and immune-cell phenotypes, provides a unifying framework linking atrial aging to AF, HFpEF, and cerebrovascular injury. Nevertheless, these markers have been evaluated across diverse experimental models and species, necessitating further validation of their specificity, translational relevance, and clinical utility. Key molecular and cellular markers associated with atrial aging are summarized in Table 4 and the Figure.

**Table 4:** Cell-type–specific aging markers and experimental contexts across atrial tissues and cell populations across species.

Experimental subjects	Aging-related markers	Organ/cells studied	Reference
Humans (18–49 vs. > 50 years old)	Proteins p53 and p21	LA	PMID: 36468256
Young SR patients (18–49 years old) vs. young AF patients (18–49 years old)	Proteins p53 and p21	LA	PMID: 36468256
AF vs. SR patients	*CAST, ASAH1, MAFB, VCAN, FTL, HEXB, PROS1, BNIP3L, PABPC1, YBX3, and S100A6*	LA	PMID: 38452746
Aged AF vs. aged non-AF patients	Elevated levels of apoptosis, ROS, fibrosis, proteins TFG-β1, Col I, Col III, Drp-1, Fis-1, reduction in proteins Pgc-1α, Mfn2, Opa1, mitochondrial complex proteins (I-V)	LA	PMID: 39499445
Patients with AF	Capillary rarefaction, increase in collagen 1	LA	PMID: 16382287
Aged humans (47.1 ± 3.3 vs. 73.4 ± 1.1 years old)	Transcript *Prom2*	RA	PMID: 38757782
Young and aged humans (14 ± 3 vs. 60 ± 3 years old)	Reduction in neuronal noradrenaline reuptake transporter (uptake 1)	RAA	PMID: 12392837
Aged humans (< 50 vs. 61–70 and >0 years old)	Increase in collagen, TFG-β, and MMMP9 at >61 years of age, reduction in Hif1α at >70 years of age	RAA	PMID: 19234766
Aged humans (44 vs. 71 and 93 years old)	SA-β-Gal activity, transcript *Cdkn2a* (p16)	hCPC isolated from AA	PMID: 29675777
AF patients	Increase in senescent CD8^+^ T cells, release of interferon-γ from these cells	LA and CD8^+^ T cells	PMID: 39382426
Aged mice (7 weeks vs. > 13 months old): C57BL/6	Fibrosis, proteins p53, p21, Col1A1, p300	Atrium	PMID: 36468256
Aged mice (5 months vs. 18 months old): C57BL/6	SA-β-Gal activity	Atrium	PMID: 36468256
Aged mice (< 20 weeks vs. > 60 weeks old)	Conduction delay in right atrium, fibrosis in LA and RA	Atrium	PMID: 34490788
Aged mice (6–8 weeks vs. > 24 months old): C57BL/6	Reduction in transcript and protein Ezh2, increase in fibrosis, and proteins Col—I, Col-III, Vimentin, p21 and p16	LA	PMID: 35996686
Aged mice (6–8 weeks vs. 24 months old): C57BL/6	Reduction in protein Exh2	LA and fibroblasts from the atrium	PMID: 35996686
Aged rats (3 vs. 20 months old): Sprague-Dawley rats	Reduction in conduction velocity, increase in APD50 (ms), increase in LA collagen, transcript and protein p16, transcripts *Csf2*, *Serpine2*, and *Mmp9*	Atrium	PMID: 38181429

AA, atrial appendages; AF, atrial fibrillation; hCPC, human cardiac progenitor cells; LA, left atrium; RA, right atrium; RAA, right atrial appendages; ROS, reactive oxygen species; SA–Gal, senescence-associated β-galactosidase; SR, sinus rhythm.

## 15 Circulating biomarkers reflecting myocardial and systemic senescence

Circulating biomarkers provide an integrated read-out of cardiac, vascular and systemic biological aging, although many of these markers are not specific to myocardial senescence and may reflect systemic inflammation, fibrosis, or general biological aging. Of these, GDF-15 has emerged as a consistently robust predictor of adverse outcomes across acute coronary syndromes, AF, and HF, reflecting oxidative stress, endothelial dysfunction, and tissue aging [102], [103], [104], [105], as summarized in a review by Candia et al. [106]. IGFBP7, a component of the senescence-associated secretome, increases in the plasma of HFrEF and HFpEF and is a strong predictor of hospitalization and mortality in HF [6], [107]. Similarly, fibrosis-linked galectin-3 predicts incident HF in population cohorts [108], while paradoxically protecting against pathological cardiac aging in knockout models, where its absence is shown to exacerbate myocardial hypertrophy, fibrosis, and apoptosis, with systemic galectin-3 KO mice exhibiting poor survival [109]. Large-scale human proteomics identify SASP proteins, including GDF15, IGFBP2, and Cystatin-C, as systemic aging biomarkers associated with metabolic dysfunction and declining physical performance [110]. Interventions, such as caloric restriction, modulate this signature by reducing circulating TNFR1/2, uPAR, MMP1, and GDF15 in middle-aged adults [111], suggesting that these markers are dynamic and modifiable. Recently, acyl coenzyme A-binding protein (ACBP) has been reported to increase in the plasma of humans with aging and to be associated with pathological aging [112].

Inflammatory cytokines, including IL-6 and TNF-α, are associated with frailty and sarcopenia in aging adults [113]. SASP factors, including GDF15, TNF receptor superfamily member 6 (FAS), TNFα, IL6, TNF receptor 1 (TNFR1), ACTIVIN A, chemokine (C—C motif) ligand 3 (CCL3), and CCL4 increase in plasma with aging, and a panel of SASP factors including osteopontin and IL-15 is associated with adverse events in patients with aortic stenosis [114]. Likewise, the level of osteopontin increases in the plasma of mice with aging and promotes myocardial fibrosis by suppressing cardiac fibroblast senescence, enhancing myofibroblast activation and ECM production, thereby contributing to age-related cardiac dysfunction [70]. Systemic immunoglobulin biology has also emerged as a senescence biomarker, with IgG accumulating in spatially defined senescence-sensitive niches and driving macrophage and microglial senescence, while its reduction ameliorates multi-organ aging phenotypes, including those in the heart in experimental models [115].

Circulating biomarkers are also reported to capture several dimensions of atrial aging. Progressive age-related increases in cardiac-derived microRNA-328 correlate strongly with AF burden in spontaneously hypertensive rats, indicating a potential role for microRNA-328 as a circulating diagnostic and potentially predictive biomarker of AF [116]. Higher pre-ablation levels of angiopoietin-2 (ANGPT2), bone morphogenetic protein-10 (BMP10), and N-terminal pro-B-type natriuretic peptide (NT-proBNP) are independently associated with early AF recurrence after first-time ablation, suggesting their potential utility in risk stratification [117]. These biomarkers reflect disturbed cardiomyocyte metabolism, altered shear stress, and increased atrial load, thus pointing to the possible role of upstream biological processes in sustaining AF despite rhythm-control therapy [117]. Cardiovascular magnetic resonance demonstrates associations between atrial dysfunction and circulating levels of soluble urokinase plasminogen activator receptor (sUPAR), galectin-3, and MMP9, highlighting inflammation and fibrosis as core pathways of atrial aging and dysfunction, although further studies are required to establish causal relationships and clinical specificity [118].

## 16 Therapies targeting senescent cells and senescence-associated secretory phenotypes

### 16.1 Senolytic strategies: eliminating senescent cardiac cells

Senolytic therapy has evolved from a conceptual framework to a diverse therapeutic class targeting senescent cardiovascular cells through immune, humoral, small-molecule, and nanotechnology-based approaches. The dasatinib–quercetin combination therapy (D + Q) is well known to mediate senolysis, and several clinical trials are underway to test its effect across multiple disorders [119], [120]. The D + Q combination has been demonstrated to eliminate senescent cardiomyocytes, cardiac stem/progenitor cells (CSCs), and non-cardiomyocytes, and has been shown to improve LV function in preclinical models after MI [121]. Similar benefits are observed in human DOX-exposed cardiac organoids, where accumulation of senescent markers and reductions in beating rate and frequency are reversed with D + Q treatment [122]. ABT-263 (Navitoclax) is another well-known senolytic that has been reported to eliminate senescent cardiomyocytes, attenuates profibrotic protein expression, and improves survival and LV systolic function after MI in preclinical models [123]. Immune engineering strategies are becoming increasingly prominent, with chimeric antigen receptor T cell platforms being adapted to selectively eliminate senescent cardiovascular and stromal cells [124]. Antigen-directed nano-vaccines may further extend this paradigm, given that the glycoprotein non-metastatic melanoma protein B (GPNMB) peptide–K36 self-assembled nano-vaccine (GK-NaV) removes GPNMB-positive senescent adipocytes and cardiomyocytes by activating dendritic cell- and CD8^+^ T cell-mediated immunity, and has been reported to improve metabolic and cardiac systolic/diastolic function, leading to a reduction in cardiomyocyte CSA in progeroid models [125]. Recent advances also emphasize precision delivery. ROS-responsive, CD9-targeted nano-assemblies have been shown to selectively deliver navitoclax to senescent cardiomyocytes, thereby attenuating senescence-associated secretory phenotype–associated remodeling in preclinical models [126]. Together, these findings highlight the therapeutic potential of senolytic strategies in cardiovascular aging, although current evidence remains largely based on animal and experimental models, underscoring the need for cautious interpretation and rigorous validation in human populations.

## 17 Senomorphic strategies: reprogramming senescence without cell elimination

Whereas senolytics have been shown to reduce senescent cell burden, senomorphics aim to suppress or reprogram the deleterious effects of senescence. β1-adrenergic blockade with metoprolol has been reported to reverse vasopressin-induced H9c2 cardiomyocyte senescence by restoring nicotinamide adenine dinucleotide metabolism, sirtuin 1 activity, and telomerase function while suppressing p53–p21 signaling [127]. Melatonin has been shown to attenuate cardiomyocyte senescence in the myocardium of type 1 diabetic mice by restoring Klotho and telomerase expression and by reducing IL-1β signaling and SA-β-Gal activity [128]. Targeting inflammatory SASP pathways has been shown to be equally effective, with the JAK1/JAK2 inhibitor ruxolitinib ameliorating diminished LV systolic function, elevated levels of p53, p21, and p16 proteins, DNA damage markers and SASP factors observed in the heart of a lipopolysaccharide (LPS)-induced murine septic cardiomyopathy model [129]. Notably, human studies have revealed sex-specific responses with short-term quercetin treatment reducing senescence markers in the internal thoracic artery (ITA) in male coronary artery disease (CAD) patients, but not in female patients [130], while also lowering the risk of new-onset AF during the postoperative in-hospital period [130]. Emerging metabolic senomorphics may further expand this class, with the microbiome-derived metabolite phenylacetic acid inducing endothelial senescence via mitochondrial hydrogen peroxide, whereas acetate has been shown to restore sirtuin 1-dependent protection [131]. Encephalopathy develops in cyclin-dependent kinase-like 5 (CDKL5) deficiency disorder (CDD), and Cdkl5^+/−^ mice exhibit QTc elongation and signs of cardiac aging, characterized by cardiac fibrosis, mitochondrial dysfunction, and high ROS levels [132]. Interestingly, coenzyme Q10 has been shown to mitigate oxidative stress in the heart of Cdkl5^+/−^ mice and reduce DNA damage in cardiomyocytes of Cdkl5+/− mice [133]. Of senescence associated with genetic diseases, TGF-β/NF-κB-associated endothelial senescence in Marfan syndrome has been reported to be reversible by pathway inhibition [134]. Autophagy-promoting interventions constitute another important class of senomorphic strategies. The natural polyamine spermidine exerts cardio-protective effects through activation of autophagy in the heart, leading to amelioration of LV diastolic dysfunction and extension of lifespan in aged mice [55]. Spermidine is essential for fasting-mediated autophagy and longevity [135]; another report has also demonstrated that spermine and spermidine delay brain aging through induction of autophagy in senescence-accelerated mice [136]. Circulating acyl-coenzyme A-binding protein/diazepam-binding inhibitor (ACBP/DBI) acts as a systemic pro-aging factor, with its neutralization preventing both natural and DOX-induced cardiomyocyte senescence while restoring autophagy, mitochondrial function/ programs, and multi-organ homeostasis [112], [137]. Extracellular vesicles from young cardiosphere-derived cells recapitulate rejuvenating effects of young blood, which include reductions in heart weight, cardiac fibrosis, cardiomyocyte size, LV diastolic dysfunction, reversing senescence signatures and extending lifespan in aged rats [138].

Together, these studies suggest that senomorphic strategies may attenuate cardiovascular aging by reprogramming senescence-associated signaling networks, including inflammatory, metabolic, mitochondrial, and autophagy-related pathways. However, much of the current evidence is derived from experimental and preclinical models, and further studies are required to determine their translational relevance, efficacy, and long-term safety in humans.

## 18 Metabolic and nicotinamide adenine dinucleotide axis rejuvenation

Nicotinamide adenine dinucleotide (NAD^+^) has crucial roles in redox homeostasis, DNA repair, and energy homeostasis [139] and declines with aging in systemic organs, including the heart [31], [140]. Nicotinamide phosphoribosyltransferase (NAMPT) plays a major role in maintaining intracellular NAD^+^ levels, and its cardiomyocyte-specific depletion results in diminished LV systolic function, increased cardiac fibrosis, and poor survival [141]. Mitochondrial dysfunction and NAD^+^ depletion are proposed as central, targetable contributors to myocardial aging. The nicotinamide adenine dinucleotide phosphate oxidase (NOX)-mimetic vanadium nanozyme has been shown to restore the NAD^+^ redox balance, suppresses cardiomyocyte senescence, and enhances post-MI repair, particularly when combined with conductive stem-cell hydrogels in preclinical models [142]. MicroRNA-based rejuvenation converges on the same axis: microRNA-203 has been shown to prevent PARP1-mediated NAD^+^ depletion, thus preserving mitochondrial function and LV diastolic performance, while the level of this microRNA declines in the myocardium with aging [140].

Collectively, these studies suggest that restoration of NAD^+^ homeostasis and mitochondrial function may represent a promising therapeutic strategy for mitigating myocardial aging, although further studies are required to establish long-term efficacy and translational applicability.

## 19 Endothelial cell, valvular cell rejuvenation, and SGLT2 inhibitors

Sodium-glucose cotransporter 2 (SGLT2) inhibitors exert direct senomorphic effects beyond glucose lowering. Of these, empagliflozin has been shown to attenuate drug-induced cardiomyocyte senescence by restoring connexin 43-dependent autophagy and suppressing inflammation-associated senescence in HFpEF models in preclinical settings [143], [144]. Given the prominent role of endothelial senescence in vascular dysfunction, multiple endothelially-targeted rejuvenation strategies have emerged. These include restoration of endothelial progenitor cell function, modulation of the cyclin-dependent kinase inhibitor 2A locus activity, regulation of metabolic and endosome/lysosomal systems, and telomerase stabilization [145], [146], [147], [148]. Advanced tools, such as circular RNA-based telomerase reverse transcriptase delivery and transient Yamanaka factor induction, are of interest, as they have been shown to reverse endothelial senescence [149], [150]. Senescence is also associated with valvular pathology. TGF-β/phosphoinositide 3-kinase (PI3K)/protein kinase B (AKT)/mammalian target of rapamycin (mTOR) signaling promotes valvular interstitial cell senescence in myxomatous mitral valve disease, and inhibition of the pathway has been shown to reverse this phenotype [151].

Together, these findings suggest that rejuvenation strategies targeting endothelial, valvular, and myocardial senescence may mitigate age-associated cardiovascular dysfunction through modulation of autophagy, telomerase activity, metabolic signaling, and pro-senescent pathways. However, most current evidence is derived from experimental and preclinical studies, and further validation is required to establish their translational relevance, efficacy, and safety in humans.

## 20 Metformin and the aging heart: metabolic reprogramming, injury modulation, and disease-specific complexity

Metformin is increasingly recognized as a modulator of cardiac aging through its effects on mitochondrial metabolism, cellular stress responses, and fibroblast activation. Metformin has been demonstrated to exert acute cardioprotective effects during reperfusion in aged hearts, with its high-dose administration at the time of reperfusion partially inhibiting mitochondrial complex I in ischemic myocardium, limiting mitochondrial permeability transition pore opening and reducing infarct size in preclinical models [152]. These benefits are accompanied by increased glycogen synthase kinase-3β phosphorylation in wild-type but not in Sestrin2-deficient mice, underscoring a Sestrin2-dependent component of metformin-mediated mitochondrial protection and highlighting the role of complex I modulation as a therapeutic strategy for reducing reperfusion injury in the aging heart [152]. Another mechanistic axis centers on succinate, which accumulates in aged myocardium and acts as a pro-fibrotic metabolite [153]. Elevated succinate activates succinate receptor 1 and triggers lysine-125 succinylation of pyruvate kinase muscle isozyme M2, thereby promoting its dimerization, nuclear translocation, and hypoxia-inducible factor-1α–dependent fibroblast activation [153]. Notably, metformin has been shown to attenuate this pathway by reducing myocardial succinate accumulation and mitigating diastolic dysfunction, thus suggesting that the succinate–pyruvate kinase muscle isozyme M2 axis is a metabolic contributor to cardiac fibrotic aging [153].

At the population level, however, the cardiovascular effects of metformin appear heterogeneous. Mendelian randomization analyses suggest that metformin use may reduce susceptibility to hypertrophic cardiomyopathy and valvular heart disease although it may paradoxically increase the risk of MI, pointing to disease-specific mechanisms that require further delineation [154].

Together, these studies position metformin as a pleiotropic modulator of cardiac aging and injury, with the potential to attenuate metabolic contributors to fibrosis, limiting reperfusion damage via mitochondrial complex I, and influencing disease susceptibility in a context-dependent manner. Its diverse mechanistic footprint reinforces the need for precision approaches that address such issues as disease substrate, age-related metabolic remodeling, and inter-individual variation in therapeutic response.

## 21 Rapalogs: mechanistic target of rapamycin inhibition as a modulator of cardiovascular and valvular aging

Rapamycin has been shown to improve impaired peripheral blood flow in aged mice and in models of atherosclerosis and Alzheimer's disease by inhibiting the mechanistic target of rapamycin (mTOR) signaling in preclinical models. These findings suggest that mTOR hyperactivation is associated with age- and disease-associated vascular dysfunction and provide a rationale for considering mTOR inhibitors as therapeutic candidates for peripheral artery disease and related circulatory disorders [155]. Loss of mitogen-activated protein kinase kinase 6 (MKK6) shortens lifespan in mice by provoking early-onset cardiac hypertrophy that progresses to dilatation and fibrosis through suppression of p38 alpha and compensatory hyperactivation of the mitogen-activated protein kinase kinase 3–p38 gamma/delta–mechanistic target of rapamycin axis [156]. Hypertrophy has been shown to be attenuated by genetic deletion of p38 gamma/delta or by rapamycin, thus suggesting that a noncanonical p38 gamma/delta pathway may contribute to pathological remodeling and represent a potential therapeutic target, with the caveat that long-term p38 alpha inhibition may be associated with cardiotoxicity [156]. In myxomatous mitral valve degeneration, senescent TGF-β1-activated valve interstitial cells display defective autophagy, while mTOR inhibition- or autophagy-related protein (ATG)-mediated autophagy induction clears p21 and p16 via sequestosome-1 (SQSTM1/p62), thus reversing senescent phenotypes and senescence-associated secretory phenotype [157]. A conceptual review has been provided to reframe cardiac nutrient signaling, demonstrating that PI3K/mTOR and sirtuin-1/adenosine monophosphate-activated protein kinase pathways exert protective or maladaptive effects depending on their signaling intensity, duration, and context, thereby defining cardiomyocyte homeostasis as a dynamically regulated network rather than a fixed program [158]. More broadly, geroscience provides a framework that seeks to modify the fundamental aging biology encompassing nutrient-sensing, proteostasis, autophagy, inflammation, and cellular senescence to extend disability-free survival. Animal and early human studies indicate that interventions, such as caloric restriction, mTOR inhibition, and senolytic strategies reshape age-related vulnerability to chronic diseases, even as their long-term clinical benefits in humans remain to be fully established, as reviewed in Kritchevsky et al. [159].

## 22 Exercise

Physical exercise is a well-established non-pharmacological intervention that preserves cardiac structure and function during aging. Emerging evidence further suggests that exercise exerts anti-senescence effects by suppressing chronic inflammation and the senescence-associated secretory phenotype, thereby addressing the fundamental hallmarks of cardiovascular aging and promoting durable cardioprotection. Telomere length shortens in the hearts of aged mice [160], while voluntary wheel-running exercise increases telomeric repeat-binding factor 1 (TRF1) and telomeric repeat-binding factor 2 (TRF2) proteins and transcripts as well as DNA repair genes, and attenuates age-associated loss of telomere length [160]. Of note, high-intensity interval training has been shown to ameliorate infarction in young and aged rat ischemia-reperfusion models [161]. Exercise also promotes the secretion of small extracellular vesicles enriched in miRNA from brown adipose tissue, and has been shown to mediate cardioprotective effects in an ischemia-reperfusion mouse model [162]. Also of interest is a report demonstrating that exercise suppresses branched-chain amino acids (BCAA) accumulation in the heart and subsequently inactivates mTOR in MI hearts, thus mediating cardioprotection [163]. Exercise further ameliorates DOX-induced cardiac systolic/diastolic dysfunction [164] and protects against DOX-induced cardiotoxicity by enhancing FcγRIIB expression in B cells, thereby promoting anti-inflammatory signaling and mitigating cardiac injury [165]. Collectively, these findings indicate that exercise has been observed to confer cardioprotective effects across diverse experimental contexts, including aging, ischemia–reperfusion injury, and cardiotoxic stress, by preserving telomere integrity, enhancing DNA repair capacity, reprogramming metabolism, and modulating inter-organ and immune signaling in preclinical models. Thus, physical activity is becoming of interest not only as a functional intervention but as a multi-system regulator of molecular resilience counteracting the fundamental mechanisms of cardiac aging and injury.

Taken together, the therapeutic strategies discussed in this section collectively suggest the potential to modulate key pathways implicated in cardiac aging. These advances open a path toward mechanism-based, chamber-specific therapeutic strategies targeting cardiac aging. Importantly, however, a substantial proportion of the supporting evidence is derived from experimental and preclinical models, and their clinical applicability requires further validation in human studies.

## 23 Conclusion and future directions

Accumulating evidence indicates that cardiac aging is not a uniform, passive process but rather a highly structured and cell-type-specific remodeling program that differentially shapes the ventricle and the atrium. In the ventricular myocardium, progressive accumulation of senescent cardiomyocytes, endothelial cells, fibroblasts, and immune cells converges on shared downstream pathways accounting for mitochondrial dysfunction, impaired proteostasis, chronic inflammation, and extracellular matrix remodeling, ultimately leading to functional decline and reduced stress adaptability. In contrast, aging of the atrium is characterized by a distinct constellation of structural, cellular, and inflammatory alterations that preferentially destabilize electrical integrity, promote fibrosis, and create a substrate particularly susceptible to AF. Together, these observations underscore that atrial and ventricular aging, while conceptually linked, are governed by partially divergent biological programs with distinct clinical consequences.

Across both chambers, cellular senescence is increasingly recognized as a key integrator of aging-associated stress, metabolic dysfunction, and inflammatory signaling. Importantly, senescence is not uniformly deleterious: transient or context-dependent senescence programs may support tissue repair and adaptation, whereas persistent accumulation of senescent cells and their associated secretory phenotypes drive maladaptive remodeling. This duality highlights the need to move beyond simplistic models of senescence as a purely pathological state and toward a more nuanced understanding of its timing, cellular origin, and functional output in the aging heart.

Several key challenges and opportunities will shape the next phase of research in cardiac aging. First, greater imaging resolution is needed to delineate chamber-specific and cell type-specific senescence programs, particularly in the atrium, where aging aligns with arrhythmogenesis, thrombogenicity, and cardiometabolic disease. Advances in single-cell and spatial multi-omics, coupled with high-resolution imaging and longitudinal human phenotyping, will be essential for mapping these programs in vivo. Second, distinguishing adaptive from maladaptive senescence states and identifying the molecular switches that govern this transition remain a critical unmet need. Third, while senolytic and senomorphic strategies show promise in experimental models, their translation will require carefully weighing their cell specificity, timing, and potential disruption of beneficial senescence-associated functions, as well as validation of efficacy and safety in human populations.

Finally, integration of circulating biomarkers, imaging-derived functional metrics, and tissue-level senescence signatures may allow for earlier detection of maladaptive cardiac aging and more precise risk stratification, particularly for AF and HF in older adults, although many of these biomarkers are not cardiac-specific and require further validation for clinical application. By unifying mechanistic insight with clinical phenotyping, future work has the potential not only to clarify how the heart ages, but also to identify rational, chamber-specific strategies for preserving cardiac function throughout lifespan. These insights collectively provide a framework for developing mechanism-based, chamber-specific interventions targeting cardiac aging.

## CRediT authorship contribution statement

**Laurent Bultot:** Writing – original draft. **Natsuko Tsurudome:** Writing – original draft. **Emi Kumazaki:** Writing – original draft. **Ippei Shimizu:** Writing – review & editing, Writing – original draft, Supervision, Conceptualization.

## Funding

This work was supported by the Intramural Research Fund for Cardiovascular Diseases of the National Cerebral and Cardiovascular Center (to I.S.).

## Declaration of competing interest

The authors declare that they have no known competing financial interests or personal relationships that could have appeared to influence the work reported in this paper.
